# The influence of D-ribose ingestion and fitness level on performance and recovery

**DOI:** 10.1186/s12970-017-0205-8

**Published:** 2017-12-20

**Authors:** John G. Seifert, Allison Brumet, John A. St Cyr

**Affiliations:** 1 Movement Science Laboratory, Montana State University, Bozeman, MT USA; 2 Jacqmar, Inc, Minneapolis, MN USA; 3 Health and Human Performance, 103E Romney Gym, Montana State University, Bozeman, MT 59717 USA; 4 Dept HHD, MSU, Bozeman, MT 59717 USA

**Keywords:** Power output, High intensity exercise, Fitness level

## Abstract

**Background:**

Skeletal muscle adenosine triphosphate (ATP) levels are severely depleted during and following prolonged high intensity exercise. Recovery from these lower ATP levels can take days, which can affect performance on subsequent days of exercise. Untrained individuals often suffer the stress and consequences of acute, repeated bouts of exercise by not having the ability to perform or recovery sufficiently to exercise on subsequent days. Conversely, trained individuals may be able to recover more quickly due to their enhanced metabolic systems. D-Ribose (DR) has been shown to enhance the recovery in ATP; however, it is not known if recovery and performance can be benefitted with DR ingestion. Therefore, this study was designed to determine what influence DR might have on muscular performance, recovery, and metabolism during and following a multi-day exercise regimen.

**Methods:**

The study was a double blind, crossover study in 26 healthy subjects compared 10 g/day of DR to 10 g/day of dextrose (DEX, control). All subjects completed 2 days of loading with either DR or DEX, followed by 3 additional days of supplementation and during these 3 days of supplementation, each subject underwent 60 min of high intensity interval exercise in separate daily sessions, which involved cycling (8 min of exercise at 60% and 2 min at 80% VO_2_max), followed by a 2 min power output (PO) test. Subjects were divided into two groups based on peak VO_2_ results, lower VO_2_ (LVO_2_) and higher peak VO_2_ (HVO_2_).

**Results:**

Mean and peak PO increased significantly from day 1 to day 3 for the DR trial compared to DEX in the LVO_2_ group. Rate of perceived exertion (RPE) and creatine kinase (CK) were significantly lower for DR than DEX in the LVO_2_ group. No differences in PO, RPE, heart rate, CK, blood urea nitrogen, or glucose were found between either supplement for the HVO_2_ group.

**Conclusion:**

DR supplementation in the lower VO_2_ max group resulted in maintenance in exercise performance, as well as lower levels of RPE and CK. Unlike no observed benefits with DEX supplementation.

## Background

It has been well established that exercise training leads to many physiological and biochemical adaptations. Proper training leads to increased glycogen synthesis, greater muscular uptake of glucose, attenuation in insulin resistance, improved redox status, and improved fat oxidation capacity in trained individuals compared to the untrained [1–7]. Because of their fitness level, untrained individuals suffer the stress and consequences of acute, repeated bouts of exercise by not having the ability to perform or recovery sufficiently to exercise on subsequent days [8, 9]. Additionally, the trained individual has the capacity to oxidize significantly more fat at the same exercise intensity compared to the untrained. This, by itself, should provide an improved recovery pattern, provided there are adequate substrates to facilitate recovery, for the trained individual due to the sparing of muscle glycogen and by providing a greater energy producing potential.

Every cell requires adequate levels of adenosine triphosphate (ATP) to maintain its energy level and function. During and following prolonged, repeated high intensity exercise, reaching or exceeding anaerobic threshold, ATP levels are depleted severely in skeletal muscle. Previous studies have demonstrated that the recovery of ATP levels can take days, which ultimately can affect performance and potentially the ability to exercise to a full extent day after day [10–12]. The recovery of ATP levels in skeletal muscle involves the adenine salvage pathway or de novo ATP synthesis. The ability to replenish this ATP deficiency following high intensity exercise is important in maintaining both the biochemical and physical cellular status following exercise [13, 14]. Therefore, it is important that the recovery of muscle ATP levels following exercise should be as quick as possible. Various substrates have been investigated to try to enhance this recovery in ATP levels.

D-ribose (DR), a naturally occurring pentose carbohydrate, has shown promise by enhancing the recovery in depressed ATP levels following stress. Due to low levels of ribose in habitual daily diets, supplementation of DR may be beneficial to produce the necessary levels of cellular energy, i.e.ATP, during and following high intensity exercise. D-ribose plays an important role as a building block for nucleotides, coenzymes, nicotinate adenine dinucleotide phosphate, nucleic acids, and ATP. Even with this ability to enhance the recovery in ATP levels, its role in aiding muscular performance following high intensity exercise and endurance has revealed mixed results [15–18]. The purpose of this project was to investigate the influence of supplementing DR ingesting on muscular performance, recovery, and exercise metabolism during and following a consecutive days of high intensity exercise. To our knowledge, this is the first study assessing the effects of DR on both high and low intensity consecutive exercise sessions.

Based on the work of Barr et al. [8] and Tomlin et al. [9], it was hypothesized that the performance and biochemical variables for the less trained subjects will be improved by the feeding of DR compared to dextrose. It is also hypothesized that the trained subjects will not gain a benefit from the supplementation of DR.

## Methods

### Subjects

After obtaining Institutional Review Board approval at Montana State University in Bozeman, MT, written informed consent was obtained from 26, healthy subjects (Table 1). The 26 subjects were divided into two equal-numbered groups based on their peak oxygen uptake (VO_2_) results, lower peak VO_2_ (LVO_2_) and higher peak VO_2_ (HVO_2_). The LVO_2_ group (females *n* = 6, males *n* = 7, average age of 27.7 ± 3.4 y) had an average peak VO_2_ of 39.9 ± 4.1 mL/kg/min. This group was involved in minimal exercise training throughout the week. The HVO_2_ group (females *n* = 4, males *n* = 9, average age of 27.6 ± 3.5 y and an average peak VO_2_ of 52.2 ± 4.3 mL/kg/min. The HVO_2_ group consisted of individuals who were consistently exercising. This high intensity exercise protocol was designed as a double blind, crossover study to assess the influence of DR (10 g/d, Bioenergy Life Sciences, Inc., Minneapolis, MN) versus control (dextrose (DEX), 10 g/d). Each subject was required to maintain their normal diet during this study, as well as performing their normal daily activities without performing any additional separate exercise sessions not designated in this study.

**Table 1:** Subject characteristics (*n* = 26)

	Age	Peak VO_2_
Females (*n* = 10)	28.0 ± 3.2	44.4 ± 6.6
Males (*n* = 16)	28.4 ± 2.7	46.8 ± 8.8

Age: years; Peak VO2: mL^.^kg^.^min^−1^

Five grams of either supplement was mixed with their food or in a self-selected beverage with lunch and an additional 5 g was ingested with dinner for 2 days as a loading dose prior to exercise. On the following 3 days of exercise, subjects ingested a standardized pre-exercise snack along with 5 g of the supplement 2 h before their exercise session. The standardized snack was self-selected, but was based on subjects’ normal dietary habits and was consistent from day to day. Following exercise, subjects ingested their final daily dose of 5 g before leaving the laboratory along with a snack which consisted of 170 g of yogurt and two granola bars.

### Experimental design

#### Pre-testing (baseline) assessment

During the first visit to the laboratory, each subject signed the informed consent and completed a health history questionnaire. Thereafter, each subject underwent a maximal oxygen uptake test and practiced the 2 min power test assessment using a cycle ergometer (Monark 834E, Sweden). Each subject completed a standardized “warm up” period at a self-selected cadence at 1 kg resistance. Cycling resistance was then increased by 0.5 kg/4 min until volitional exhaustion. Heart rate (HR), VO_2_ and a blood lactate (Lac) sample were collected at 3:30 of each 4:00 stage. This assessment established exercise workloads during the two treatment sessions.

The exercise session consisted of six, 10 min intervals of exercise on a cycle ergometer. Each interval required the subject to cycle for 8 min at a workload that corresponded to about 60% of their peak VO_2_ followed by 2 min at one workload above their calculated lactate threshold (approximately 80% peak VO_2_). At the end of the 60 min exercise session, each subject completed a 2 min performance task. This performance task required the subject to produce as much power as possible during this 2 min interval. Peak power (PO) and average power was assessed during this 2 min task trial. Workload for this test was set at 5% of their body weight. A minimum of 4 day washout period was employed between crossover arms of the study to allow adequate recovery.

#### Biochemical and physiological measurements

Venous blood samples were drawn via a venipuncture 15 min before exercise and 24 h post exercise. Finger stick techniques at 10 min before exercise, 20 min, 40 min, and 60 min during exercise. The finger stick blood samples collected during exercise were assayed for blood glucose. Creatine kinase (CK) and blood urea nitrogen (BUN) concentrations were measured at pre-exercise and 24 h post exercise following the last exercise session. Subjects ingested 200 ml of water at 20 and 40 min during exercise to minimize any effects of dehydration.

Rating of perceived exertion (RPE) was recorded every 20 min during exercise using the Borg 1-10 scale. Heart rate was recorded using a Polar HR monitor (RC3; Polar, Inc., Finland). Blood glucose (Gluc) concentration were measured using a Bayer Gluc monitor (Bayer Medical, NJ). Blood lactate levels were measured by an AccuSport Lactate Analyzer (Akira, Japan). Creatine kinase and BUN were measured utilizing an Abaxis Piccolo analyzer (Princeton, NJ). Power data from the time trial performance test was assessed with the Sports Medicine Industries (SMI) software package (St.Cloud, MN).

### Statistical analysis

Statistical analyses of performance, physiological measurements, and laboratory values during and following intense exercise provides evidence of what specific role DR can play in a untrained or trained athlete. Data are presented as mean ± SD. Data was analyzed with SPSS statistical software using a 2-way ANOVA with repeated measures with time and treatments as independent variables. A Turkey’s post hoc test was used to differentiate means if a significant interaction was observed. Heart rate, RPE, blood Gluc, serum CK, serum BUN and power data were dependent measures. Analysis data is represented as mean ± SD and an alpha level of significance was set at *p* < 0.05.

## Results

All 26 subjects completed the study without any adverse events. Data are presented as main effects as there were no interactions. No statistical differences were observed for the dependent measures when data were analyzed from 26 subjects. Therefore, subjects were divided into two equal groups based on their VO_2_max values.

Relative and absolute mean power data can be found in Table 2. D-ribose ingestion led to a significant (*p* = 0.04) improvement in relative mean power over DEX in the LVO_2_ group. There was also a significant difference between DR and DEX in the change of absolute mean power (*p* = 0.01) for this group. Significant differences between DR and DEX were found for relative (*p* = 0.05) and absolute (*p* = 0.02) peak PO for the LVO_2_ group. The average changes in relative and absolute peak power from Day 1 to Day 3 were 0.33 ± 0.52 W/kg BW and 26.8 ± 40.8 W for DR while DEX were −0.09 ± 0.51 W/kg BW and −10.8 ± 33.0 W, respectively.

**Table 2:** Changes in relative and absolute mean power output from day 1 to 3

	Lower VO_2_ group	Higher VO_2_ group
Ribose		
Relative	0.17 (0.32)*	0.08 (0.39)
Absolute	13.2 (24.2)**	2.3 (17.1)
Dextrose		
Relative	−0.09 (0.29)	0.07 (0.33)
Absolute	−8.9 (22.4)	8.2 (27.7)

Mean (± SD); Relative power: W/kg BW; Absolute power: W

*significantly different from Dextrose (*p* = 0.04)

**Significantly different from Dextrose (*p* = 0.01)

Relative and absolute mean PO were not different between DR and DEX treatments for the HVO_2_ group. No differences between treatments were noted for relative (*p* = 0.27) and absolute (*p* = 0.79) peak PO for the HVO_2_ group. The average changes in relative and absolute peak PO from Day 1 to Day 3 were 0.15 ± 0.41 W/kg BW and 6.2 ± 28.6 W for DR while DEX were −0.02 ± 0.37 W/kg BW and 3.31 ± 25.8 W, respectively.

Analysis of serum CK data indicated that DR ingestion led to lower change in the LVO_2_ group. Creatine kinase levels increased by an average of 37.1 ± 85.2 U/L for the DR treatment compared to the DEX treatment of 121.4 ± 110.2 U/L (*p* = 0.03). No statistical difference (*p* = 0.88) was observed for change in BUN levels between DR (0.93 ± 2.66 mM/L) and DEX (1.08 ± 2.56 mM/L) treatments for the LVO_2_ group. No differences for change in CK and BUN levels were observed between DR (54.1 ± 243 U and 2.2 ± 2.4 mM/L) and DEX (49.5 ± 226 U and 0.9 ± 3.7 mM/L) treatments in the HVO_2_ group. No differences were observed for blood Gluc and remained stable for all treatments and within both groups (Table 3).

**Table 3:** Blood glucose response during exercise

	Lower VO_2_			Higher VO_2_		
	Time (min)			Time (min)		
	19.5	39.5	59.5	19.5	39.5	59.5
Ribose	4.0 (0.6)	4.0 (0.6)	4.1 (0.7)	3.8 (0.5)	4.0 (0.5)	3.9 (0.5)
Dextrose	4.0 (0.5)	4.0 (0.5)	3.9 (0.6)	4.0 (0.6)	4.1 (0.7)	4.0 (0.6)

Mean (± SD); Values in mM/L

No difference between treatments was found for HR in the LVO_2_ group. Average HR for the DR trial was 152 ± 20 bpm and 153 ± 17 bpm for the DEX trial. Rating of perceived exertion was significantly lower (*p* = 0.003) for DR (13 ± 2) than DEX (14 ± 2) in LVO_2_ group. Average HR and RPE across the exercise days were not different between DR and DEX for the HVO_2_ group, 153 ± 12 bpm and 14 ± 2 versus 153 ± 12 bpm and 14 ± 2, respectively.

## Discussion

The purpose of this project was to investigate the influence of DR on muscular performance and recovery during and following a multi-day high intensity exercise regimen in LVO_2_ and HVO_2_ groups. The pertinent results indicate that DR ingestion improved performance and recovery for the LVO_2_ group during a multi-day exercise study, but not in the HVO_2_ group.

The role in which DR could play on performance during intense exercise has been uncertain. Due to this uncertainty, this study has demonstrated that DR supplementation provided a performance benefit undergoing repeated days of high intensity exercise. The rate of ATP utilization during high intensity exercise may exceed ATP production and a considerable amount of time is required for these deficient levels to recover [10, 19]. Hellsten et al. [20] reported in subjects supplementing with oral DR around high-intensity, intermittent exercise showed that ATP synthesis increased and ATP levels return to normal after 72 h; however, this benefit was not found in the control group. A decrease in cellular high energy phosphates can affect muscular function besides producing symptoms of soreness and fatigue, which can affect subsequent exercise sessions [21]. Studies have revealed mixed results in replenishing muscular energy levels, maintaining or enhancing performance and alleviating post-exercise symptoms with substrate supplementation [22].

Alternatives to metabolic pathways can play important roles in ATP generation, such as the pentose phosphate pathway. The pentose phosphate pathway is critical for the formation of 5-phosphyl-ribose-1-pyrophosphate (PRPP), an intermediate in the production of ATP, and plays a role in ATP de novo synthesis which is dependent on a rate limiting enzyme, glucose 6-phosphte dehydrogenase (G6PDH) [21]. However, DR is unique in that it bypasses this rate limiting enzymatic step in the formation of PRPP [23]. This enhanced production of ATP by DR replenishes the cellular energy deficiency and can shorten the energy recovery period following high intensity exercise. It is plausible that subjects in the present study’s HVO_2_ group, had well developed energy-producing systems that when stressed, were able to overcome the exercise stress through other various recovery processes. On the other hand, subjects in the LVO_2_ group may not have had the ability to fully utilize other pathways (i.e. PRPP) to assist in recovery. Thus, DR gave them extra substrate to bypass the G6PDH step and, potentially, increase the efficiency of the recovery, reflecting a potential increase in muscle ATP levels. This study did not measure muscle ATP levels, which could have provided additional supporting data for both the trained and untrained athlete. The measurement of muscle ATP levels could provide a more in-depth metabolic explanation.

The effect of DR on performance has provided mixed results. Raue et al. [18] reported that 220 g of DR supplementation (20 g/d) produced a significant increase in average power output during high intensity exercise. Berardi et al. [24] found that DR supplementation prior to exercise produced an increase in peak PO from 2.2 to 7% and increased their average PO from 2 to 10%. Van Gammerren et al. [17] reported that 280 g of DR supplementation (10 g/d) increased muscular power assessment in weight lifting increased muscular strength and total work performed in amateur bodybuilders. However, DR has not always demonstrated an improvement in performance. Eijnde et al. [15] found that the use of 16 g/d of DR during knee contraction exercise did not produce a benefit in PO nor in ATP synthesis in physically active subjects. Furthermore, Kreider et al. [16] reported that supplementing 10 g/day for 5 days of DR around exercise did not demonstrate a significant difference from control subjects when undergoing anaerobic exercise capacity test in trained subjects, as well as not reflecting an improvement in metabolic parameters.

The present study demonstrates that subject selection criterion (i.e. fitness level) has a significant influence in the results. When data were analyzed by treatment (DR vs. DEX), regardless of fitness level, no statistical differences were observed. In fact, values were comparable between the treatments. Untrained individuals appear to suffer the consequences of acute, repeated bouts of exercise by not having the ability to perform or recovery sufficiently to exercise on subsequent days [8, 9]. The potential beneficial role of DR also depends upon the dosage and timing of dosing, type of exercise, degree of intensity and duration of exercise. We designed a high-intensity exercise protocol where cellular anaerobic metabolism commences; thereby stressing the metabolic activity in these exercising muscles and to see what role DR may play on recovery and performance. In evaluating performance in the LVO_2_ group, we found that mean and peak PO increased significantly with DR from day 1 to 3, which was not observed in the DEX treatment. Multiple factors can account for the performance benefits with DR. For example, differences in muscular CK levels might shed light on this beneficial difference in performance by indicating a maintenance, or lack thereof, of cell membrane integrity. The change in CK level from day 1 to day 3 was about 3 times greater for the DEX treatment compared to DR in the LVO_2_ group. This could indicate that there was a mechanism by which the cells were able to recover energy for the next day’s exercise and be able to continue to demonstrate at least an adequate level of performance in that group. Besides the impact of high intensity exercise on cellular metabolism, additional factors may also play a role, such as reactive oxygen species.

High intensity exercise may result in oxidative damage in both the blood and skeletal muscle [25]; however, high intensity exercise is superior to low intensity exercise in upregulating the muscle to produce superoxide dismutase and GSH peroxidase [26]. Seifert et al. [27] reported that DR ingestion led to significantly lower production of free radical markers compared to the control treatment during exercise under hypoxic conditions. The biochemical mechanisms responsible for these symptoms remain unclear; however, the production of free radicals could play an important role as mediators of muscular damage. Sjodin et al. [19] reported that during exercise, two potentially harmful free radical sources are mitochondrial semiquinone and xanthine oxidase in the endothelial cells. The metabolic stress during exercise alters the biochemical state of the cell, which ultimately enhances the rate of oxygen free radical production from semiquinone and xanthine oxidase. It is therefore, plausible that if mitochondrial function is altered during exercise, performance may be inhibited. This study did not measure produced products of oxidative stress, which could have provided additional interesting and supporting data during and following high intensity exercise.

The delivery and utilization of oxygen to exercising muscle is a major factor in assessing fitness and maximal VO_2_ levels [28]. Upon further assessment of our subjects in this study into LVO_2_ and HVO_2_ groups, revealed significant differences when consuming DR during the high intensity exercise sessions for the LVO_2_ group. These findings appear to suggest that individuals that have not consistently performed exercise above the Lac threshold level do not fair equally with individuals that exercise or train on a more intense regimen schedule. The rise in CK levels observed in the LVO_2_ group appears to imply that a strenuous, anaerobic exercise produced cellular stress in which enzymatic leaking occurs, which can not only effect cellular homeostasis, but performance and recovery as well.

## Conclusions

In conclusion, high intensity, anaerobic exercise decreases muscular ATP levels and a considerable amount of time is required for these lower energy levels to recover. Some studies have reported mixed performance benefits with DR, probably reflecting protocol differences, dosing of DR, timing of the DR dosage, intensity of exercise, and subject specificity. For this last reason we developed a protocol that induced a level of high intensity, anaerobic exercise in two fitness level groups. The analysis revealed that the LVO_2_ value subjects had a significant improvement in performance, lower changes in CK, and lower RPE with DR compared to DEX. Assessment of metabolic serum parameters did not reflect any appreciable differences between the treatments, not clearly demonstrating a potential mechanism accounting for this benefit. A limitation of this study would be dietary control. While subjects were instructed to maintain their dietary habits as usual during the study, an in depth analysis of intakes was not performed. It is possible that some members of the LVO2 group had an insufficient diet that would have benefitted from supplementation. In summary, DR demonstrated a performance, perceptual, and serum benefits in the lower fitness adult subjects undergoing high intensity exercise. The stress of high intensity exercise has the potential to be benefited with supplementation of DR around these exercise sessions. Future studies are needed to elucidate the mechanism(s) of action of DR ingestion and exercise.
